# Empirical Comparison and Analysis of Artificial Intelligence-Based Methods for Identifying Phosphorylation Sites of SARS-CoV-2 Infection

**DOI:** 10.3390/ijms252413674

**Published:** 2024-12-21

**Authors:** Hongyan Lai, Tao Zhu, Sijia Xie, Xinwei Luo, Feitong Hong, Diyu Luo, Fuying Dao, Hao Lin, Kunxian Shu, Hao Lv

**Affiliations:** 1 Chongqing Key Laboratory of Big Data for Bio Intelligence, Chongqing University of Posts and Telecommunications, Chongqing 400065, China; laihy@cqupt.edu.cn (H.L.); s240502011@stu.cqupt.edu.cn (T.Z.); s230502010@stu.cqupt.edu.cn (D.L.); 2 Center for Informational Biology, School of Life Science and Technology, University of Electronic Science and Technology of China, Chengdu 611731, China; a994318971@163.com (S.X.); suziqingcoin@163.com (X.L.); ftong_hong@163.com (F.H.); hlin@uestc.edu.cn (H.L.); 3 School of Biological Sciences, Nanyang Technological University, Singapore 639798, Singapore; fuying.dao@ntu.edu.sg

**Keywords:** SARS-CoV-2, phosphorylation site, machine learning, deep learning, computation tool

## Abstract

Severe acute respiratory syndrome coronavirus 2 (SARS-CoV-2) is a member of the large coronavirus family with high infectivity and pathogenicity and is the primary pathogen causing the global pandemic of coronavirus disease 2019 (COVID-19). Phosphorylation is a major type of protein post-translational modification that plays an essential role in the process of SARS-CoV-2–host interactions. The precise identification of phosphorylation sites in host cells infected with SARS-CoV-2 will be of great importance to investigate potential antiviral responses and mechanisms and exploit novel targets for therapeutic development. Numerous computational tools have been developed on the basis of phosphoproteomic data generated by mass spectrometry-based experimental techniques, with which phosphorylation sites can be accurately ascertained across the whole SARS-CoV-2-infected proteomes. In this work, we have comprehensively reviewed several major aspects of the construction strategies and availability of these predictors, including benchmark dataset preparation, feature extraction and refinement methods, machine learning algorithms and deep learning architectures, model evaluation approaches and metrics, and publicly available web servers and packages. We have highlighted and compared the prediction performance of each tool on the independent serine/threonine (S/T) and tyrosine (Y) phosphorylation datasets and discussed the overall limitations of current existing predictors. In summary, this review would provide pertinent insights into the exploitation of new powerful phosphorylation site identification tools, facilitate the localization of more suitable target molecules for experimental verification, and contribute to the development of antiviral therapies.

## 1. Introduction

Severe acute respiratory syndrome coronavirus 2 (SARS-CoV-2) is a novel positive-strand RNA beta-coronavirus with a genome size of ~30 kb [1]. Since its emergence at the end of 2019, the rapid spread of SARS-CoV-2 has caused coronavirus disease 2019 (COVID-19), severely impacting global human health and social public security. The typical clinical characteristics of COVID-19 include fever, dry cough, and fatigue, which can progress to acute respiratory distress, hyperinflammation, respiratory failure, multiple organ failure, and even death [2,3].

Protein phosphorylation, a well-studied type of post-translational modification (PTM), involves the addition of phosphate groups to specific amino acid residues [4], such as serine (S), threonine (T), and tyrosine (Y), catalyzed by protein kinases. Studies have shown that the infection mechanism of SARS-CoV-2 involves multi-level changes, with alterations in the host phosphoproteome playing a critical role [5,6,7,8,9,10,11]. For instance, Bouhaddou et al. [6] conducted a quantitative phosphoproteomics survey of Vero E6 cells infected with SARS-CoV-2 and revealed the dramatic remodeling of phosphorylation profiles on host cells and SARS-CoV-2 proteins. Similarly, Stukalov et al. [5] transduced A549 lung carcinoma cells with SARS-CoV-2 to explore the influences of SARS-CoV-2 infection on host RNA, protein, phosphorylation, and ubiquitination profiles. These findings underscore the importance of accurately identifying phosphorylation sites to unravel the complex molecular mechanisms underlying the cellular responses to viral infection, with potential implications for discovering novel therapeutic targets [12,13,14,15,16,17].

Over the past decade, mass spectrometry (MS)-based proteomics has significantly advanced our understanding of PTMs and increased the known PTM types to over 700 [18]. MS-based proteomics enables global quantification of protein abundance and phosphorylation levels, providing an effective approach for analyzing the changes in protein and phosphorylation profiles of hosts induced by SARS-CoV-2 infection [19,20]. However, these methods often suffer from either low throughput or excessive labor demands and prohibitive costs. Consequently, in silico techniques, characterized by their efficiency and precision, have emerged as a supplementary tool for identifying novel modifications [21]. To date, many computational approaches have been developed to predict phosphorylation sites [22], yet systematic reviews summarizing state-of-the-art methods for identifying phosphorylation sites in SARS-CoV-2 infections are still lacking.

**Figure 1:**
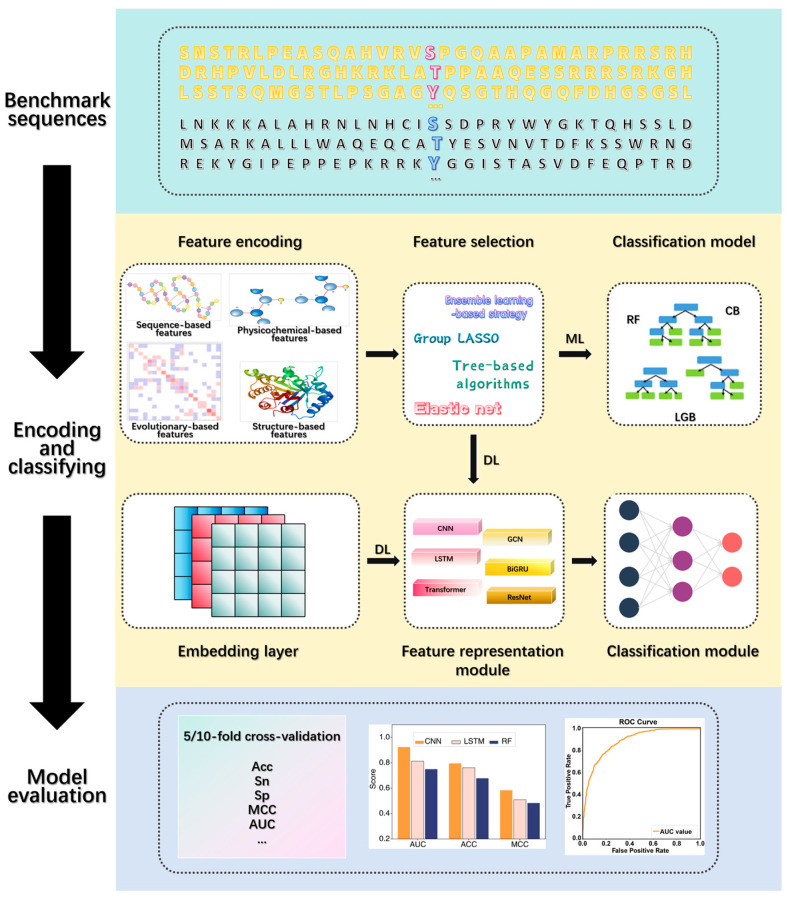
The workflow of existing computational tools for predicting phosphorylation sites in host cells infected with SARS-CoV-2. These tools are developed based on conventional machine learning models and end-to-end deep learning networks, mainly through the following steps: benchmark sequence data preparation, feature encoding and selection, classification model design, and prediction assessment.

To fill this gap, we conducted a comprehensive outline of the computational models for specifically identifying protein phosphorylation modification sites in host cells infected with SARS-CoV-2. This review focuses on key aspects such as benchmark dataset construction (data collection and preprocessing), feature encoding and selection strategies, classification model design, and prediction assessment and comparison (Figure 1). For classification model design, we summarize two main strategies: traditional machine learning (ML)-based methods and end-to-end deep learning (DL)-based frameworks. Lastly, we provide a detailed comparison of published methodologies and accessible tools. We believe this review offers valuable insights for advancing research in viral physiology and biology while serving as an effective recommendation system for subsequent experimental validation.

## 2. Materials and Methods

### 2.1. Benchmark Datasets of Phosphorylation Sites in SARS-CoV-2-Infected Host Cells

**Figure 2:**
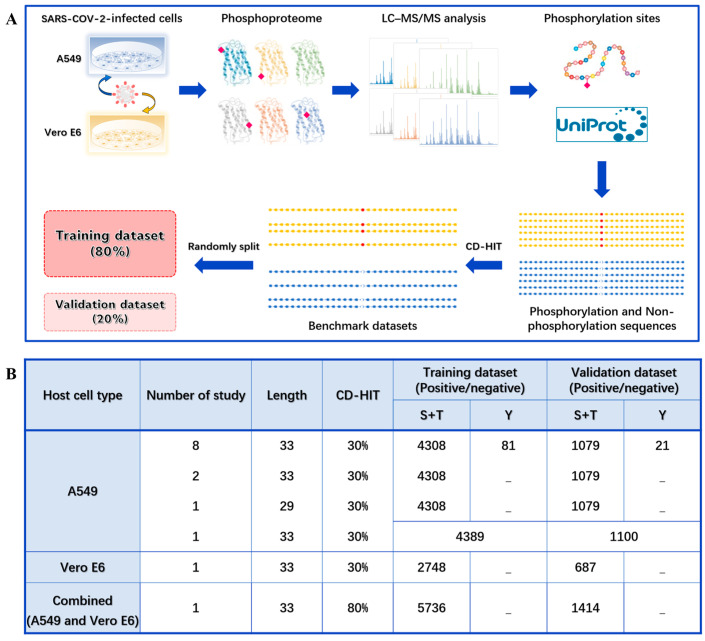
The well-studied benchmark datasets of phosphorylation sites in host cells infected with SARS-CoV-2. (**A**) The originations, as well as the common collection and preprocessing procedures of benchmark datasets. (**B**) The detailed information of the three benchmark datasets, named A549 (human), Vero E6 (African green monkey), and combined.

High-quality benchmark datasets are the key component in constructing computational tools with strong predictive performance [23,24,25]. Here, we introduce the data resources and preprocessing pipeline for SARS-CoV-2-infected phosphorylation sites. Overall, the primary data sources for all related studies are two datasets: the Vero E6 dataset and the A549 dataset. The former was generated based on the global phosphorylation profile of SARS-CoV-2-infected African green monkey Vero E6 cells (PRIDE ID: PXD019113) [6], while the latter was obtained based on the abnormal landscape of protein and phosphorylation in human lung-originated A549 cell line resulting from SARS-CoV-2 infection (PRIDE ID: PXD022282) [5] (Figure 2).

Following similar workflows (Figure 2A), Lv et al. and Pham et al. independently created the A549 benchmark dataset and the Vero E6 benchmark dataset for model construction [26,27]. First, the positions of phosphorylation sites and protein annotations were obtained from processed MS data downloaded from the PRIDE database. Second, protein sequences were downloaded from UniProt database to construct positive samples centered on phosphorylated S/T/Y sites, with 16 upstream and 16 downstream amino acid residues [18]. Accordingly, sequence segments of 33 residues centered on non-phosphorylated S/T/Y sites were extracted as immature negative samples. Third, the CD-HIT program, with a 30% threshold, was used to cluster protein sequences to eliminate redundancy and avoid model overfitting [28]. Fourth, immature negative samples were randomly downsampled to obtain mature negative samples matching the number of positive samples. Finally, the A549 benchmark dataset contained 5387 positive and 5387 negative samples, while the Vero E6 benchmark dataset contained 3435 positive and 3435 negative samples. Pham et al. [27] further integrated these two benchmark datasets into one dataset, named combined, which contained 6790 positive and 6790 negative samples. Detailed information for each dataset is shown in Figure 2B.

### 2.2. Feature Extraction Strategies

Feature encoding based on sample sequences is an essential step for the development of phosphorylation site predictors using conventional ML models or even DL models [29,30,31,32,33,34]. More than twenty feature extraction algorithms have been employed in the prediction of phosphorylation sites in SARS-CoV-2-infected host cells, which are focused on capturing the primary amino acid composition, physicochemical property, evolution, and structure information (Table 1). The brief description of these methods is as follows:

(1) Amino acid composition (AAC)

AAC is the simplest protein sequence-based feature, which encodes a protein or a segment as a numerical vector with 20 factors. Each factor means the frequency of each type of amino acid residue in the corresponding sequence.

(1) $$AAC=\frac{N\left(p_{i}\right)}{L};0<i\leq 20$$

where L is the length of a sequence (namely 33 in this survey), and $N\left(p_{i}\right)$ is the number of i-th type amino acid residue in the sequence.

(2) Dipeptide composition (DPC)

DPC is a feature extraction method that characterizes the types and relative abundance of consecutive amino acid pairs in a sequence. There are 400 possible dipeptides, each formed by two specific amino acids [35].

(2) $${DPC}_{\left(l,r\right)}=\frac{N_{lr}}{N-1},l\in \left\{A,R,D,...,Y\right\}$$

where l and r represent the left and right amino acids of a dipeptide, respectively. $N_{lr}$ is the number of dipeptides composed of amino acids l and r, an N is the total number of amino acids in the protein or peptide sequence. This calculation results in a 400-dimensional vector, which captures the relative frequencies of all possible dipeptides, thereby providing a detailed representation of the dipeptide composition within the sequence.

(3) Amino acid index (AAI)

AAindex is a comprehensive database that provides 20 numerical values representing the physicochemical and biological properties of amino acids [36]. This database includes 566 indices, of which 553 are valid (excluding NaN values), offering extensive information about each amino acid within a sequence. To generate meaningful features, these indices are averaged across the sequence using the following formula:

(3) $$AAI\left(j\right)=\frac{\sum_{i=1}^{N}AAI\left(A_{i}\right)}{L}$$

in which the j represents the 553 indices in the AAindex database, $A_{i}$ denotes the amino acid at position i in the sequence, and L is the total number of residues in the sequence. This calculation captures the averaged physicochemical and biological properties of amino acids, enabling detailed characterization of the sequence [37].

(4) Composition–transition–distribution (CTD)

CTD is a method used to comprehensively characterize the physicochemical properties of amino acids in peptide or protein sequences [38]. It consists of three components: composition (CTDC), transition (CTDT), and distribution (CTDD). The CTDC measures the frequency of amino acids belonging to specific property groups (e.g., positive, neutral, and negative) within a sequence. The CTDT captures the frequency of transitions between different property groups, such as a positive residue followed by a neutral one. The CTDD evaluates the distribution of residues with specific properties at defined intervals, specifically in the first 25%, 50%, 75%, and 100% of the sequence.

Using the CTD method, a 21-dimensional feature descriptor is generated for each amino acid property. By leveraging 13 distinct physicochemical properties, this method produces 39-dimensional features for CTDC and CTDT and a 195-dimensional feature for CTDD, resulting in a comprehensive representation of sequence properties.

The formulas for CTDC, CTDT, and CTDD components are as follows:

(4) $$CTDC\left(x\right)=\frac{N_{x}}{L}$$

where $N_{x}$ is the count of residues with property x, and L is the sequence length.

(5) $$CTDT\left(x,y\right)=\frac{T_{xy}}{T_{total}}$$

where $T_{xy}$ represents the number of transitions between amino acid categories x and y in the sequence, and $T_{total}$ is the total number of transitions between all amino acid categories in the sequence. This formula calculates the transition frequency between two specific amino acid categories relative to the total transitions in the sequence, providing insight into the sequence-order information of the peptide.

(6) $$CTDD\left(x,p\right)=\frac{N_{x}^{p}}{L}$$

where $N_{x}^{p}$ is the count of residues with property x in a specific percentage p (e.g., 25%, 50%, 75%, or 100%). CTD is an alignment-free method whose accuracy depends on the classification of amino acids into property groups, such as positive, neutral, and negative. It captures both local and global sequence features, providing a detailed characterization of amino acid properties.

(5) Composition of K-spaced amino acid group pairs (CKSGP)

The CKSGP method calculates the frequency of amino acid pairs separated by k residues and categorizes these pairs into five distinct groups based on the physicochemical properties of their constituent amino acids. It extends traditional k-spaced amino acid pair composition by incorporating both sequence and physicochemical property information, providing a more detailed characterization of the regions surrounding post-translational modification sites. The CKSGP feature is calculated using the following formula:

(7) $$CKSGP=\frac{N_{p_{i}p_{j}}}{L},p_{i},p_{j}\in \left\{A,C,D....,Y\right\}$$

where $N_{p_{i}p_{j}}$ represents the count of residue pairs $p_{i}p_{j}$, and L denotes the total length of the amino acid sequence. Additionally, j=i+k+1, i,j≤L. In this method, k can range from 0 to 10, allowing the capture of amino acid pair frequencies across various distances. CKSGP also classifies dipeptide pairs (DPCs) into 25 distinct property-based classes, generating 25 descriptors for each amino acid pair. This procedure results in a 275-dimensional feature vector, offering a comprehensive representation of the sequence composition and properties.

(6) K-spaced conjoint triad (KSC)

KSC is a feature descriptor that analyzes the frequency of specific triad combinations formed by amino acids at fixed intervals in a protein sequence. The triad is defined as:

(8) $$T_{i}=\left\{A_{i},A_{i+k+1},A_{i+2k+2}\right\}$$

where $A_{i}$ is the i-th amino acid, $A_{i+k+1}$ is the amino acid located k+1 positions after $A_{i}$, and $A_{i+2k+2}$ is the amino acid located 2k+2 positions after $A_{i}$. Based on the physicochemical properties, all amino acids are divided into seven classes. Each amino acid $A_{i}$ belongs to a specific class $C_{j}$ (j=1,2,…,7). Accordingly, for a given k (the number of intervals between amino acids), there are 343 different types of triads and the KSC method will generates a vector F with 343 unique features:

(9) $$F=\left[F\left(T_{1}\right),F\left(T_{2}\right),...,F\left(T_{343}\right)\right]$$

Each feature represents the frequency of a particular triad. The frequency $F_{\left(T\right)}$ of a triad $T=\left\{C_{1},C_{2},C_{3}\right\}$ is given by:

(10) $$F_{\left(T\right)}=\frac{Count\left(T\right)}{L-k-2}$$

where $Count\left(T\right)$ is the number of occurrences of triad T in the entire sequence, and L is the length of the amino acid sequence. This method effectively can capture the spatial and physicochemical relationships among amino acids in a sequence, providing a comprehensive representation of sequence features.

(7) GXPC

GXPC is a feature vector derived by classifying the 20 amino acids based on their physicochemical properties [39] and calculating the frequencies of dipeptide and tripeptide combinations. The amino acids are classified into five categories, according to their physicochemical properties: aliphatic (IMGAVL), aromatic (FWY), positively charged (HKR), negatively charged (EDG), and neutral (QNSTCP). Based on these classifications, the dipeptide group (GDPC) can be divided into 25 categories, forming a 25-dimensional feature vector. Similarly, the tripeptide group (GTPC) can be divided into 125 categories, generating a 125-dimensional feature vector. Finally, the 25-dimensional GDPC and 125-dimensional GTPC vectors are linearly combined to form a 150-dimensional feature vector called GXPC.

The dipeptide frequency is calculated as follows:

(11) $$F\left(Dipeptide\right)=\frac{Count\left(Dipeptide\right)}{L-1}$$

where L is the sequence length, and Count(Dipeptide) is the occurrence count of a specific dipeptide combination in the sequence. The frequencies of all 25 dipeptide combinations are computed and ordered to form a 25-dimensional feature vector.

The tripeptide frequency is calculated as:

(12) $$F\left(Tripeptide\right)=\frac{Count\left(Tripeptide\right)}{L-2}$$

where L is the sequence length, and Count(Tripeptide) is the occurrence count of a specific tripeptide combination. The frequencies of all 125 tripeptide combinations are calculated and ordered to form a 125-dimensional feature vector.

The final GXPC feature vector is obtained by directly combining the 25-dimensional GDPC and 125-dimensional GTPC feature vectors, as follows: $GXPC=\left[GDPC,GDPC\right]$. This 150-dimensional feature vector can effectively represent the structural and functional information of a peptide sequence.

(8) XPAAC

XPAAC is a method that integrates pseudo-amino acid composition (PAAC) and amphiphilic pseudo-amino acid composition (APAAC) to provide a comprehensive representation of both local and global sequence information for proteins or peptides. This approach combines the frequency-based encoding of amino acids and the autocorrelation of physicochemical properties, resulting in a unified 55-dimensional feature vector.

The PAAC component captures local sequence information by calculating the frequency of each amino acid in the sequence, producing a basic 20-dimensional vector. The frequency is computed as:

(13) $$f_{i}=\frac{\mathrm{Count}\left(i\right)}{L}$$

where $\mathrm{Count}\left(i\right)$ is the occurrence count of amino acid i in the sequence, and L is the total sequence length.

The sequence pattern features, specifically the physicochemical property autocorrelation, are computed for each position in the sequence using the following formula:

(14) $$\Theta_{k}=\frac{1}{L-k}\sum_{i=1}^{L-k}\left(P_{i}-\bar{P}\right)\left(\left(P_{i+k}-\bar{P}\right)\right)$$

where $P_{i}$ is the physicochemical property value of the i-th amino acid, and $\bar{P}$ is the mean value of that physicochemical property across the sequence. The lag distance k takes values k=1,2,…,λ (where λ=5).

APAAC describes the distribution of hydrophilicity and hydrophobicity across amino acids. It is based on the autocorrelation of these properties [40]. The global property autocorrelation is computed as:

(15) $$\Theta_{k}^{APAAC}=\frac{1}{L-k}\sum_{i=1}^{L-k}\left(H_{i}-\bar{H}\right)\left(H_{i+k}-\bar{H}\right)$$

where $H_{i}$ represents the hydrophilicity or hydrophobicity value of the i-th amino acid, and $\bar{H}$ is the global mean of the property. The lag parameter k is again set to λ=5.

The PAAC (with 35 dimensions) and APAAC (with 20 dimensions) features are combined into $XPAAC=\left[PAAC,APAAC\right].$ This 55-dimensional feature effectively encodes the structural, functional, and physicochemical properties of protein or peptide sequences, integrating both local and global sequence characteristics.

(9) Dipeptide deviation from the expected mean (DDE)

Dipeptide deviation from the expected mean (DDE) is a feature descriptor used to quantify the deviation of observed dipeptide frequencies from their theoretical expected values [41]. By considering the codon diversity and dipeptide composition, DDE provides a more refined representation of sequence characteristics. It generates a 400-dimensional feature vector by standardizing dipeptide frequencies using z-score normalization. The calculation of DDE involves the following components:

(16) $$DDE=\frac{{DPC}_{\left(l,r\right)}-{MT}_{\left(l,r\right)}}{\sqrt{{VT}_{\left(l,r\right)}}}$$

where ${DPC}_{\left(l,r\right)}$ is the frequency of dipeptide combinations in the sequence and is defined as:

(17) $${DPC}_{\left(l,r\right)}=\frac{N_{lr}}{L-1},l\epsilon \left\{A,R,D,...,Y\right\}$$

where l and r represent the left and right amino acids of the dipeptide, $N_{lr}$ is the number of dipeptides encoded by amino acids l and r, and L is the total number of amino acids in the protein or peptide sequence.

${MT}_{\left(l,r\right)}$ is the expected mean frequency of the dipeptide l, r, calculated by:

(18) $${MT}_{\left(l,r\right)}=\frac{C_{A_{l}}\times C_{A_{r}}}{C_{L}^{2}}$$

where $C_{A_{l}}$ and $C_{A_{r}}$ are the codon numbers encoding the left and right amino acids of the dipeptide, and $C_{L}$ is the total number of codons, excluding the three stop codons.

${VT}_{\left(l,r\right)}$ is the variation term, defined as:

(19) $${VT}_{\left(l,r\right)}=\frac{{MT}_{\left(l,r\right)}\times \left(1-{MT}_{\left(l,r\right)}\right)}{L-1}$$

This approach allows DDE to capture deviations between observed and expected dipeptide frequencies in protein or peptide sequences, offering a more detailed and statistically robust feature representation for sequence analysis.

(10) Quasi-sequence-order (QSO)

QSO is a feature descriptor that integrates both the normalized frequency of individual amino acids and the sequence-order coupling effects to capture local and global peptide sequence information [42]. It incorporates sequence-order effects and the physicochemical properties of amino acids to generate a 100-dimensional feature vector. The QSO feature is calculated as:

(20) $${QSO}_{i}=\frac{f_{i}}{\sum_{i=1}^{20}f_{i}+\alpha \sum_{d=1}^{nlag}\beta_{d}},i=1,2,...,20$$

where $f_{i}$ is the normalized frequency of amino acid i,α is a tuning weight factor (default value α = 0.1), d is the distance between two amino acids (d can be any integer between 1 and 20), and nlag is the maximum lag between two amino acids. $\beta_{d}$ is the sequence-order coupling number for the d-th order, defined as:

(21) $$\beta_{d}=\sum_{i=1}^{N-d}{\left(d_{i,i+d}\right)}^{2},d=1,2,3,\ldots ,nlag$$

where N is the total number of amino acids in the sequence, and $d_{i,i+d}$ represents the coupling between the i-th and $\left(i+d\right)$-th amino acids in the sequence. The QSO descriptor is obtained by combining the physicochemical properties and sequence-order effects, resulting in a vector that comprehensively represents both the local and global structural features of the peptide sequence.

(11) A combination of Shannon entropy, Geary autocorrelation, Sequence-order coupling number, and Moran autocorrelation (EGSM)

EGSM is a combination of four components: Shannon entropy, Geary autocorrelation, sequence-order-coupling number, and Moran autocorrelation. The Shannon entropy for each sequence within the dataset is determined using the following function:

(22) $$H\left(X\right)=-\sum \left(P\left(x\_y\right)*\log_{2}\left(P\left(x\_y\right)\right)\right)$$

in which y represents the 20 amino acids, and $P\left(x\_y\right)$ indicates the probability of a specific amino acid occurring within the sequence.

Geary’s and Moran’s autocorrelation descriptors are built upon the arrangement and distribution of amino acid properties sourced from AAIndex along the sequence. Prior to computing these descriptors, all indices undergo standardization. The SOCN is derived from a distance matrix for all 20 amino acids. Two distance matrices are employed, based on the work of Grantham [43] and Schneider-Wrede [44], respectively.

(12) Atomic and bond composition (ABC)

ABC is a characteristic descriptor used to analyze protein or peptide sequences. It quantifies the combined total of atomic and bond compositions for each amino acid sequence, capturing both the elemental composition and the distribution pattern of bonds. The atomic characteristics of a peptide sequence refer to the relative abundance of five elements (C, H, N, O, and S).

The formula for calculating the atomic characteristics is presented below. For each common element (C, H, N, O, and S), the formula to determine their relative abundance is as follows:

(23) $$f\left(A\right)=\frac{Count\left(A\right)}{Total\;Atoms}$$

where $f\left(A\right)$ represents the relative proportion of element A in the sequence, $Count\left(A\right)$ is the total number of element A in the sequence, and TotalAtoms is the total number of all atoms in the sequence (including all C, H, N, O, and S).

Bond characteristics include the total number of bonds, the number of single bonds and double bonds, which describe the quantity and categories of bonds in the sequence. The ABC feature vector is the combination of all elements.

(13) Alignment-based encoding of secondary structure neural network (AESNN)

AESNN uses artificial neural network to compare protein structures and uses three-dimensional vectors to represent each amino acid residue. These vectors are derived from the outputs of three hidden units in a neural network. For a given peptide sequence, AESNN generates a L × 3-D feature vector. The three-dimensional characteristic formula of amino acids is:

(24) $$h\left(xi\right)=\sigma \left(W\cdot x_{i}+b\right)$$

where $x_{i}$ is the feature vector of each amino acid, which is derived from the information after structural comparison. These features are obtained by protein comparison or database annotation. The weight matrix W and the bias vector b are determined by the trained neural network and are used to learn the three-dimensional representation activation function σ of each amino acid from the comparison features, usually ReLU or sigmoid, to transform the result of linear combination into nonlinear features, to represent the three-dimensional feature vector of each amino acid in the output $h\left(xi\right)$, and to reflect its function and network characteristics in the sequence and structure context. For a sample sequence with a length of L, the feature vector of the whole sequence is as follows:

(25) AESNN=[h(x1),h(x2),…,h(xL)]

(14) Binary 3-bit (Bit3) and Binary 5-bit (Bit5)

Binary 3-bit is a method for representing protein sequences based on binary coding that combines three amino acids together, such as {a1, a2, a3}, and each combination is represented by a 3-D binary vector. For instance, a1, a2, and a3 are encoded as (100), (010) and (001), respectively. This code classifies amino acids based on their physical and chemical properties, including hydrophobicity and other more detailed properties such as secondary structure, normalized van der Waals volume, polarizability, polarity, charge, and solvent accessibility [45]. These attributes are used to differentiate between subtypes 1 through 7. By linearly combining all seven types of features, Bit3 generates a feature with 3×7×L factors.

Binary 5-bit integrates two ways to encode protein sequences. In the first approach, known as Binary_5bit_Type 1, peptide sequences are represented by five amino acid groups {a1, a2, a3, a4, a5}, each associated with a unique 5-D binary vector. These groups, based on physicochemical properties, are encoded as follows: a1 (S, T, C, P, N, and Q) as (10000), a2 (F, Y, and W) as (01000), a3 (R, K, and H) as (00100), a4 (D and E) as (00010), and a5 (G, A, V, L, M, and I) as (00001). The second approach, Binary_5bit_Type 2, explores the 32 possible arrangements of ones and zeros within a five-bit unit, discarding configurations with no ones, all ones, or those with either 1 or 4 ones. Twenty representations are obtained for the 20 amino acids. For instance, A is encoded as (0, 0, 0, 1, 1), C as (0, 0, 1, 0, 1), and so on. By combining these two ways, the Bit5 strategy will generate a comprehensive feature vector with 5×2×L elements.

(15) Overlapping property feature (OPF)

The overlapping property feature descriptor represents protein sequences by systematically categorizing amino acids into seven groups based on their physical and chemical properties [46,47]. All amino acids are classified into three subtypes based on hydrophobicity or polarity (subtype 1), secondary structures like α helix and β sheet (subtype 2), and more nuanced properties such as van der Waals volume, charge, and solvent accessibility (subtype 3). OPF describes each protein fragment with a comprehensive feature vector with 7×3×L elements by integrating these individual subtypes.

(16) Binary profile (BINA) or binary encoding (BE)

Binary profile (also called binary encoding, BE) is a feature extraction method based on binary encoding designed to represent the identity of each amino acid residue with a 0 or 1 in a protein sequence. This method maps each amino acid residue to a 20-dimensional binary vector to characterize its features. For a single amino acid, its binary encoding is defined as:

(26) $$B\left(A\right)=\left(b_{1},b_{2},\ldots ,b_{20}\right),b_{i}=\left\{\begin{array}{c} 1,\;if\;A\;is\;the\;\mathrm{i-th}\;amino\;acid\\ 0,\;others\;\;\;\;\;\;\;\;\;\;\;\;\;\;\;\;\;\;\;\;\;\; \end{array}\right.$$

where B(A) represents the binary encoding of amino acid A, and i denotes the index of A in the 20 standard amino acids. Eventually, for a protein sequence with length L, BE feature is $BE=[B\left(A_{1}\right),B\left(A_{2}\right),\ldots B\left(A_{L}\right)]$ and the feature dimension is 20×L.

(17) Z-scale (ZSC)

Z-scale is a set of physical and chemical descriptors of amino acids proposed by Sandberg et al. in 1998 [48]. It uses five different physical and chemical properties to encode amino acid sequences, Z1 hydrophobicity, Z2 polarity or volume, Z3 electron effect, Z4 secondary structure tendency, and Z5 solvent accessibility, and finally generates a 165-D feature vector. Each amino acid is expressed as a 5-dimensional vector $Z\left(A\right)$:

(27) $$Z(A)=(Z_{1},Z_{2},Z_{3},Z_{4},Z_{5})$$

$Z_{1},Z_{2},Z_{3},Z_{4},andZ_{5}$ represent five physicochemical property values of the amino acid, which have been normalized by Sandberg et al. [48] For a sequence S with a length of L: $S=\left\{A_{1}{,A}_{2},...,A_{L}\right\}$, the dimension of ZSC is L×5, which is expressed as:

(28) $$ZSC=\left[Z\left(A_{1}\right),Z\left(A_{2}\right),\ldots Z\left(A_{L}\right)\right]$$

(18) BLOSUM62 (BLOS)

BLOSUM62 is a substitution matrix based on protein sequence alignment used to analyze the similarity and conservatism of protein sequences [49]. Based on the BLOSUM62 matrix, a given peptide sequence with L amino acids can be encoded into a feature vector with a fixed dimension of 20×L.

(19) A linear combination of enhanced AAC and enhanced Grouped AAC (EXAC)

The EXAC feature extraction method generates a high-dimensional feature vector by combining enhanced AAC (EAAC) and enhanced grouped AAC (EGAAC). It integrates the amino acid frequency derived from sliding windows and physicochemical properties based on amino acid grouping, resulting in a 725-dimension feature vector.

The EAAC calculates local amino acid frequencies using a sliding window method, with the feature dimension determined by the window size.

(29) $$f_{i}=\frac{Count\left(A_{i}\right)}{Window\;Size}$$

where $A_{i}$ is the i-th amino acid, $Count\left(A_{i}\right)$ is the number of occurrences of $A_{i}$ within the window, $f_{i}$ is the frequency of $A_{i}$, and WindowSize is the fixed length of the window. The feature dimension of EAAC is $20\times K_{1}$, where $K_{1}$ is the number of sliding windows.

The EGAAC classifies amino acids into G groups based on physicochemical properties (e.g., hydrophobicity, polarity, and charge) and calculates the local frequency of each group.

(30) $$f_{w}(G_{j})=\frac{Count(G_{j}in\;window\;w)}{L_{w}},j=1,2,\ldots ,G$$

where G is the number of amino acid groups, $f_{w}(G_{j})$ is the frequency of group $G_{j}$ within window w, and $Count\left(G_{j}in\;window\;w\right)$ is the number of amino acids in window w belonging to group $G_{j}$. The feature dimension of EGAAC is $G\times K_{2}$, where $K_{2}$ is the number of sliding windows.

The EXAC feature vector is formed by combining the EAAC and EGAAC vectors linearly, integrating local amino acid frequencies and group-based frequencies into a single feature set. The total feature dimension of EXAC is $20\times K_{1}+G\times K_{2}$.

(20) 188D

The 188D method was designed by Cai et al. to capture the physicochemical properties of amino acids in a protein sequence, which combines amino acid composition (AAC) and physicochemical property descriptors (CTD) to generate a 188-dimensional feature vector for characterizing protein sequences [50]. The first 20 features are from AAC. The remaining 168 features are calculated based on the CTD method for eight physicochemical properties of amino acids, including hydrophobicity, normalized van der Waals volume, polarity, polarizability, charge, surface tension, secondary structure, and solvent accessibility.

(21) Enhanced grouped amino acid composition (EGAAC)

EGAAC calculates the grouped amino acid composition (GAAC) within a fixed-length sliding window (default length of 5) that moves sequentially from the N-terminus to the C-terminus of each sequence:

(31) $$f_{\left(gp,w\right)}=\frac{N_{\left(gp,w\right)}}{N\left(w\right)},\;gp\in \left\{gp1,gp2,...,gp5\right\},w\in \left\{w1,w2,...,w17\right\}$$

where $N_{\left(gp,w\right)}$ represents the count of amino acids belonging to group gp within the sliding window w, and $N\left(w\right)$ denotes the size or length of the sliding window w.

(22) Grouped Tripeptide Composition (GTPC)

GTPC (grouped tripeptide composition) produces a 125-dimensional vector by computing the occurrence of grouped tripeptides in a protein or peptide sequence, with these tripeptides being classified into five groups based on their physicochemical characteristics. GTPC can be computed as follows:

(32) $${GTPC}_{\left(l,m,r\right)}=\frac{N_{lmr}}{L-2},l,m,r\in \left\{G_{1}{,G}_{2},G_{3},G_{4}{,G}_{5}\right\}$$

where $G_{1}{,G}_{2}{,G}_{3}{,G}_{4}{,\;and\;G}_{5}$ are GAVLMI (aliphatic), FYW (aromatic), KRH (positively charged groups), DE (negatively charged groups), and STCPNQ (uncharged groups), respectively. l,m,r mean the left, middle, and right amino acids of a tripeptide; $N_{lmr}$ is the number of tripeptides encoded by the amino acid types l,m,r; and L is the total number of amino acids in the protein or peptide sequence.

(24) One-hot encoding

One-hot encoding is a method for converting categorical data into a binary vector. Each category is represented by a vector of length equal to the number of possible categories, where all elements are 0 except for a single 1 that represents the specific category.

The feature vector for each element is defined as:

(33) $$x_{i}=\left\{\begin{array}{c} 1,\;if\;A\;is\;the\;\mathrm{k-th}\;type\;of\;amino\;acid\;\\ 0,\;others\;\;\;\;\;\;\;\;\;\;\;\;\;\;\;\;\;\;\;\;\;\;\;\;\;\;\;\;\;\;\;\; \end{array}\right.$$

where k denotes the category, and i is the position of the element. For a sequence of length L, the final feature vector has a dimension of L×C, where C is the number of categories.

(25) Adapted normal distribution bi-profile Bayes (ANBPB)

The adapted normal distribution bi-profile Bayes (ANBPB) algorithm represents the data in a normal distribution format and employs Bayesian inference to ascertain the unknown parameters [51]. The random variable is independent and follows a binomial distribution b(n,p), where p = 1/20 is the probability of each type of amino acid and n denotes the twenty amino acids. The standard normal distribution random probability $x_{ij}^{\prime }$ can be calculated as:

(34) $$x_{ij}^{\prime }=\frac{x_{ij}-np}{\sqrt{v_{j}}}$$

where ij indicates the i-th amino acid at the j-th position, $v_{j}$ is the standard deviation, and $p_{j}$ is the posterior probability:

(35) $$p_{j}=P\left(X\leq X_{ij}\right)=\xi \left(x_{ij}^{\prime }\right)$$

$\xi \left(x\right)$ follows a standard normal distribution, $\xi \left(x\right)=\frac{1}{\sqrt{2\pi }}\int_{-\infty }^{x}e^{-\frac{t^{2}}{2}}dt$. For protein sequences with a length of L, the final ANBPB feature vector has a dimensionality of 2×L.

(26) Pseudo-amino acid composition (PseAAC)

PseAAC (pseudo-amino acid composition) introduces a number of additional pseudo-amino acids, which are utilized to gather more comprehensive information about the protein sequence [52]. These pseudo-amino acids are developed based on properties, structures, or other distinct features of amino acids.

(36) $$PseAAC={\left[m_{1},m_{2},...,m_{20},m_{20+1},...{,m}_{20+\lambda }\right]}^{T}\left(\lambda <L\right)$$

where the $\left[m_{1},m_{2},...,m_{20}\right]$ and $\left[m_{20+1},...,m_{20+\lambda }\right]$ represent the amino acid composition and positional information in the protein sequence, respectively. L denotes the number of amino acids in the sample. Each feature component in m is defined as:

(37) $$P_{t}=\left\{\begin{array}{c} \frac{f_{t}}{\sum_{t=1}^{20}f_{t}+\omega \sum_{i=1}^{\lambda }\tau_{i}}\;\;\;\;\;\;\;\;\;\;\;\;\;\;\;\;\;\;\;\;\;1\leq t\leq 20\\ \frac{\omega \tau_{t-20}}{\sum_{t=1}^{20}f_{t}+\omega \sum_{i=1}^{\lambda }\tau_{i}}\;\;\;\;20+1\leq t\leq 20+\lambda \end{array}\right.$$

where $f_{t}$ represents the probability of the occurrence of the type t amino acid in the sample, ω is the weight factor, and $\tau_{i}$ denotes the sequence COR relationship factor. The feature vector extracted by PseAAC has a dimensionality of 20+λ.

### 2.3. Conventional Machine Learning Algorithms

On the basis of the encoded numerical vector features, twelve conventional ML algorithms had been tried to classify phosphorylation sites in host cells infected with SARS-CoV-2, including random forest (RF) [53], extremely randomized trees (ERT), gradient boosting trees (GBT), extreme GBT (XGBT), AdaBoost (AB), catBoost (CB) [54], light GBT (LGBT) [27], k-neighbors classifier (KNN), support vector machine (SVM), logistic regression (LR), multilayer perceptron (MLP), Gaussian naïve Bayes (GNB) [55]. Among them, the tree-based models were the most commonly used, and RF, CB, and LGBT were separately employed by three studies to build corresponding final optimal classifiers.

(1) Tree-based classifier

Tree-based classification models are a type of supervised learning algorithm that rely on tree-like structures, primarily used for classification tasks. They recursively partition data into different subsets to form a decision tree, thereby achieving data classification. Each internal node represents a decision rule for a feature, while split points signify thresholds or conditions, and each leaf node corresponds to a classification label.

Decision tree is a machine learning model based on a tree structure that recursively splits the feature space to construct decision rules for classifying or regressing data. Similar to a tree structure, decision tree comprises a root node, several internal nodes, and several leaf nodes. Leaf nodes correspond to decision outcomes, while each other node corresponds to an attribute test. The sample set contained in each node is divided into child nodes based on the attribute test results. The root node contains the entire sample set, and the path from the root node to each leaf node corresponds to a sequence of decision tests. Similarly, the process of generating a decision tree model is also recursive.

RF, ERT, GBT, XGBT, AB, CB, and LGBT are ensemble learning models that use decision trees as individual learners. Based on how individual learners are generated, they can be classified into two categories: boosting and bagging (bootstrap aggregating). The boosting algorithms build strong models through gradual optimization, which focuses on improving accuracy by iteratively constructing multiple weak learners in a weighted manner. Each new round of model construction primarily focuses on data that performed poorly in the previous round, including AB (adjusting sample weights to gradually increase attention to misclassified samples), GBT (gradually optimizing the loss function by fitting residuals), XGBT (improving the GBT algorithm by incorporating optimization methods such as regularization and parallelization, enhancing performance and efficiency), LGBT (also based on GBT optimization and using histogram-based split algorithms and a leaf-wise growth strategy to optimize training speed and memory efficiency), and CB (a boosting algorithm optimized for categorical features and focusing more on reducing gradient bias). The bagging algorithm enhances model stability through parallelized sampling by randomly sampling data multiple times, training multiple weak learners, and combining their outputs (such as through voting or averaging) to form the final prediction. RF is also based on decision trees with strong resistance to overfitting, which constructs multiple trees on random samples using the bagging strategy to form an ensemble model. ERT is an improved version of RF that adds random split points on top of RF to further improve model diversity, randomness, and training speed.

(2) Other ML classifiers

KNN is a distance-based classification algorithm. Its basic principle is to calculate the distance between a test sample and all samples in the training set, select the K nearest neighbor samples, and determine the classification result of the test sample based on the majority category of these samples. It does not require explicit training and is suitable for small-scale data, but it has high computational complexity when processing high-dimensional data.

SVM is a classification algorithm based on the maximum margin, aiming to find an optimal hyperplane that separates samples of different categories while maximizing the minimum distance from the samples to the hyperplane. For nonlinear classification problems, SVM uses kernel functions to map data into a high-dimensional space to find a linearly separable classification hyperplane in that space.

LR is a linear classification algorithm that models the relationship between sample features and categories as a linear equation by constructing a log-odds function and uses a sigmoid function to map the result to a probability value. It is suitable for linearly separable binary classification problems, is simple and efficient, and has good interpretability.

GNB is a generative classification algorithm based on Bayes’ theorem, assuming that features are independent of each other and that each feature follows a Gaussian distribution under the condition of a category. It calculates the posterior probabilities of each category and selects the category with the highest probability as the classification result. GNB is simple to implement and suitable for small-scale data.

MLP is a feedforward neural network that achieves nonlinear mapping of features through multiple hidden layers. Each layer transforms the input using a weighted sum and activation function to extract complex features layer by layer. MLP is good at capturing complex nonlinear correlations and is suitable for complex tasks such as image and speech processing.

### 2.4. Deep Learning Networks

End-to-end deep learning has emerged as a trending topic in the realm of ML, captivating the ML community [56,57]. Among all DL networks, convolutional neural network (CNN), long short-term memory (LSTM), and bidirectional gate recurrent unit (BiGRU) are the most famous, and they are also used more frequently in studies aimed at recognizing phosphorylation sites in host cells infected with SARS-CoV-2 [26,58,59]. In addition, the transformer architectures based on self-attention or multi-head mechanisms, as well as graph convolutional network (GCN) and residual network (ResNet), widely used in the field of computational identification of PTMs [60], have also been employed to develop phosphorylation site predictors [61,62].

CNNs are neural networks specifically designed in deep learning to process data with grid-like topological structures, such as images and time series. Inspired by the processing mechanisms of biological visual systems, CNNs simulate the local connections and feature-sharing properties among neurons to achieve efficient feature extraction and pattern recognition. The core components of CNNs include convolutional layers, activation functions, pooling layers, and fully connected layers. Their hierarchical structure enables the network to progressively extract information from local low-level features to global high-level features. GCN [63] is a deep learning model similar to CNN, which is designed for graph data. GCNs can effectively capture the relationships between nodes and local structures by performing convolutional operations on graph structures, thereby enabling feature learning and prediction for graph data. Similar to the multi-layer convolution in CNNs, GCN can extract features from local to global levels layer by layer.

LSTM is primarily used to address the issue of long-term dependencies in recurrent neural networks (RNNs), and it is a special type of RNN. Conventional RNNs may fail to capture long-term dependencies in long sequence training due to the vanishing or exploding gradient problem. However, LSTM effectively mitigates this issue by introducing a gating mechanism. LSTM lies in its unique gate structure, which, through the interaction of multiple gates, flexibly controls the storage, updating, and output of information. In virtue of the gating mechanism, LSTM can effectively capture dependencies in long time series, automatically selecting the information to store or forget and adapting to the needs of various tasks. However, it suffers from limitations in processing long sequence data owing to the high computational complexity and vanishing and exploding gradients problems.

BiGRU [64] is an upgraded extension of the gated recurrent unit (GRU), specifically designed to capture the bidirectional dependencies in sequence data. The unidirectional GRU addresses the vanishing gradient problem of ordinary RNNs by introducing gating mechanisms such as the update gate and reset gate, but can only utilize historical information from the sequence and cannot capture future information. To address this issue, BiGRU consists of two GRU layers processing the sequence in both forward and backward directions, which can comprehensively capture contextual features by simultaneously leveraging both directions of information and is suitable for tasks with strong contextual relevance.

Transformer is a deep learning architecture entirely based on the attention mechanism, designed to capture the dependencies between different positions in a sequence. By integrating position encoding modules, multi-head attention mechanism, et al., it can capture global contextual information and achieve efficient parallel computation. Transformer is adaptable to varying input sequence lengths, making it suitable for a wide range of tasks, including text, image, and biological sequence processing. The multi-head attention mechanism is the core module of transformer architectures, tasked with capturing the dependencies between different positions in the input sequence. By computing in parallel across multiple attention heads, it can extract features from different subspaces and capture important information within the input sequences, thereby providing enhanced expressive power and bolstering the model’s capacity to learn complex patterns.

ResNet is a deep neural network that addresses the issues of gradient vanishing and gradient exploding in the training of deep networks by introducing residual connections. It simplifies the training process and enhances model performance by learning the residual between the input and the target output, rather than directly learning a complex mapping function. ResNet can stack numerous layers to improve the model’s representation capacity while reducing the learning difficulty of deep networks by learning residuals.

### 2.5. Model Evaluation

To comprehensively evaluate the predictive performance of a classification model, apart from assessing its prediction accuracy on the validation dataset, K-fold cross-validation and a series of evaluation metrics are typically employed as well.

In some studies on phosphorylation site predictor development, both 5-fold and 10-fold cross-validation have been applied in both ML/DL model training and validating processes. The K-fold cross-validation [65] is a resampling procedure involving splitting the whole dataset into K equal subsets, which can take advantage of limited data sets. In this process, the model is trained and validated K times; for each time, a different subset is selected as the validation set, while the remaining K−1 subsets are used to train model. Through K iterations, performance evaluation results of K validation sets are obtained, and the average of these results is usually calculated to estimate the overall performance of the model [66].

Moreover, K-fold cross-validation is often used in combination with other evaluation measures and methods to provide a more comprehensive landscape of model performance, such as accuracy (Acc), sensitivity (Sn), specificity (Sp), Matthews correlation coefficient (MCC), AUC (area under ROC, the receiver operating characteristic curve) and AUPR (area under PRC, the precision–recall curve). The specific calculation formulas are as follows:

(38) $$\left\{\begin{array}{c} Acc=\frac{TP+TN}{TP+FN+TN+FP}\\ Sn=\frac{TP}{TP+FN}\\ Sp=\frac{TN}{TN+FP}\\ MCC=\frac{TP\times TN-FP\times FN}{\sqrt{\left(TP+FP\right)\times \left(TP+FN\right)\times \left(TN+FN\right)\times \left(TN+FP\right)}} \end{array}\right.$$

TP is short for true positive, which means the number of phosphorylation sequences that the model correctly predicted to be positive. FN stands for false negative, namely, the number of phosphorylation sequences incorrectly predicted as negative. TN (true negative) and FP (false positive) are the numbers of non-phosphorylation sequences correctly classified as negative samples and incorrectly classified as positive samples, respectively. MCC and AUC (balancing Sn and Sp), as well as AUPR (balancing precision and recall), are commonly used statistical indices to evaluate the performance of a binary classification model. The value range for MCC is [−1, 1], and for both AUC and AUPR is [0, 1]. The closer MCC, AUC, and AUPR values are to 1, the better is the performance of prediction models [67,68].

## 3. Phosphorylation Site Predictors Based on Conventional ML Models

In this section, we reviewed five predictors designed with conventional ML algorithms for identifying phosphorylation sites in host cells infected with SARS-CoV-2 (Table 2).

Liu et al. proposed a RF-based phosphorylation site predictor called EnsembleML [53]. For feature engineering, they utilized a feature fusion approach based on 188D, AESNN, CKSAAP, and Z-scale descriptors. Using an ensemble learning strategy based on KNN, SVM, LR, MLP, GNB, and DTC, they refined these features and integrated into the RF algorithm. Finally, the top 35, 23, 139, and 122 most effective features among 188D, AESNN3, CKSAAP, and Z-scale features were selected, and their accuracy values on S/T and Y data were 76.04%, 74.42%, 72.20%, and 72.47%, and 92.86%, 76.19%, 83.35%, and 88.10%, respectively. These optimal features were directly combined to build EnsembleML. Independent verification results showed that EnsembleML could predict SARS-CoV-2-infected S/T and Y phosphorylation sites with Acc of 80.81% and 95.24%, respectively. EnsembleML effectively leveraged multiple feature selection strategies to integrate feature information, enhancing the generalization ability of the model. Moreover, its simplicity and low computational cost make it easily adaptable to other datasets. However, it is important to note that RF algorithms may be limited by the “curse of dimensionality” when dealing with high-dimensional data, especially in large-scale proteomics, where nonlinear features might not be fully captured.

Pham et al. explored the performance of the A549, Vero E6, and combined benchmark datasets in phosphorylation site identification. Specifically, they constructed three S/T phosphorylation site predictors named MeL-STPhos_1, MeLSTPhos_2, and MeL-STPhos_3 [27]. To comprehensively capture multimodal modification features, they evaluated the predictive performance of 22 feature descriptors (AAC, DPC, AAI, CTDC, CTDT, CTDD, CKSGP, KSC, GXPC, XPAAC, DDE, QSO, EGSM, ABC, AESNN, Bit3, Bit5, OPF, BINA, ZSC, BLOS, and EXAC) using 14 distinct ML classifiers (RF, CNN, DNN, CNNLSTM, ERT, MLP, SVM, GBT, XGBT, AB, CB, LGBT, DT, and LR) on different datasets. The results indicated that these feature descriptors and classifiers performed differentially on different datasets. Notably, the LGBT model based on EXAC achieved the highest Acc, with a value of 82.5%, on the A549 dataset, the XGBT-EXAC model achieved the highest Acc, with a value of 83.7%, on the Vero E6 dataset, and the AB-EXAC model had the best performance on the combined dataset with an Acc of 80.6%. After detailed feature and ML model assessment, the 22 best baseline models of each benchmark dataset were determined for the investigation of the meta-learning approach. Finally, six tree-based algorithms were utilized to select important features and build baseline models: AB, LGBT, CB, GBT, and RF for MeL-STPhos_1; AB, XGBT, GBT, LGBT, and CB for MeL-STPhos_2; and RF, XGBT, AB, LGBT, and CB for MeL-STPhos_3. Additionally, CB was applied to build the final meta-learning model for the three predictors. In terms of model performance, MeL-STPhos_1 accurately identified S/T phosphorylation sites in SARS-CoV-2-infected A549 cells, achieving Acc, MCC, and AUC values of 83.0%, 0.661, and 0.912, respectively. MeL-STPhos_2 predicted S/T phosphorylation sites in Vero E6 cells infected with SARS-CoV-2 with Acc, MCC, and AUC values of 83.6%, 0.673, and 0.913, respectively. MeL-STPhos_3 performed relatively poorly on the combined dataset, with Acc, MCC, and AUC values of 70.1%, 0.405, and 0.763, respectively.

MeL-STPhos demonstrated how to select appropriate features and models in a multi-dataset environment while optimizing prediction performance through meta-learning. However, the significant performance decline of MeL-STPhos_3 when handling combined datasets highlights the impact of cross-dataset feature distribution differences on model performance. This suggests that relying solely on feature engineering and tree-based ML models may not sufficiently capture complex feature interactions. Furthermore, the reduced performance of the combined dataset model limits its potential for integrating data from different experimental conditions or biological contexts.

Sabir et al. compared the discriminative power of ten features derived from amino acid composition, evolutionary information, and position-specific information using five ML methods, including SVM, RF, MLP, LGB, and LR [55]. Results showed that EGAAC and BLOS were the top high discriminative features, which achieved MCC values greater than 0.6 based on the LGB classifier. Furthermore, the fusion of EGAAC and BLOS features achieved the best performance, with an Acc of 83.1%, and LGB exhibited a superior prediction capability than the remaining four models. Cross-validation results on independent datasets demonstrated that LGB-IPs effectively predicted S/T/Y phosphorylation sites in host cells infected with SARS-CoV-2, achieving Acc, MCC, and AUC values of 82.6%, 0.653, and 0.905, respectively. Overall, LGB-IPs introduced a novel perspective by incorporating joint prediction of multiple residue types, offering a comprehensive approach to studying phosphorylation modifications. The advantages of the LGB algorithm in handling nonlinear features and large-scale datasets further enhanced the model’s robustness. While integrating S/T/Y information is a valuable attempt, such unified modeling may overlook the specificity of different residues in phosphorylation mechanisms. Moreover, the generalization ability of the model has yet to be validated across species or in other viral infection contexts.

## 4. Phosphorylation Site Predictors Based on DL Models

In this section, we summarized the computational tools developed based on DL algorithms for predicting phosphorylation sites in host cells infected with SARS-CoV-2. Current methods primarily employ two protein sequence encoding strategies (Figure 1, Table 2). One category adopts the fully end-to-end approach, which directly encodes phosphorylation and non-phosphorylation sequences through embedding layers and feeding them into DL network layers. Another category involves feature engineering to convert protein sequences into numerical vectors, followed by DL frameworks. In addition, different DL architectures have their own advantages in the task of predicting phosphorylation sites. CNNs possess powerful feature extraction capabilities, which can capture local sequence features by sliding the convolution kernel on the sequence and gradually learn global sequence features by stacking multiple convolutional layers. LSTMs can learn long-term dependencies in sequence data and capture contextual information in the sequence. Transformers capture the dependencies between different positions in the sequence through a self-attention mechanism and have efficient parallel computing capabilities. Theoretically, phosphorylation sites are often associated with specific sequence patterns or features and may be affected by multiple positions in the sequence, as well as by sequence elements distant from them. For encoding phosphorylation sequences more comprehensively and improving prediction performance, several tools have been designed on the strategy of hybridizing different DL networks.

(1) End-to-end deep learning methods

Lv et al. proposed the first deep learning model for predicting phosphorylated S/T or Y sites in SARS-CoV-2-infected human host cells, named DeepIPs [26]. They represented amino acid sequences using both a supervised embedding layer and unsupervised embedding layers (Word2Vec, GloVe, and fastText). By combining CNN and LSTM layers, a hybrid DL architecture was constructed. The results showed that DeepIPs achieved Acc, MCC, and AUC values of 80.63%, 0.632, and 0.894 for S/T phosphorylation site identification on the validation dataset. For Y phosphorylation site identification, DeepIPs achieved higher values of Acc, MCC, and AUC at 83.33%, 0.718, and 0.925, respectively. Based on the benchmark phosphorylation sites and corresponding phosphorylated proteins, Lv et al. [26] further analyzed related kinases and found that viral proteins accelerate the host cell cycle by virtue of interacting with the kinases of host cells. It suggested that developing specific inhibitors for these kinases could be a promising strategy to treat the SARS-CoV-2 infection.

Zhang et al. proposed a hybrid DL architecture named IPs-GRUAtt, which incorporates BiGRU networks and an attention mechanism to capture the positional information of amino acid sequences [69]. This model implemented the Keras embedding layer [70] for sequence encoding and was trained separately on the S/T phosphorylation site dataset and the Y phosphorylation site dataset. The results based on 5-fold cross-validation showed Acc, MCC, and AUC values of 84.62%, 0.632, and 0.919 for S/T phosphorylation site identification, and 92.86%, 0.858, 0.921 for Y phosphorylation site identification.

DeepIPs and IPs-GRUAtt leverage end-to-end architectures, reducing reliance on manual feature design. The combination of CNN and LSTM effectively captures both local and global sequence information, while the attention mechanism in IPs-GRUAtt further enhances interpretability.

(2) Feature engineering combined with deep learning

Jiao et al. developed a computational model named PSPred-ALE based on a self-adaptive learning embedding algorithm for recognizing SARS-CoV-2-infected S/T phosphorylation sites [61]. Additionally, they systematically compared self-adaptive embedding features with feature-engineered models. The results from 5-fold cross-validation demonstrated that PSPred-ALE achieved an AUC value of 0.907 for S/T phosphorylation site identification.

To maximize feature representation and stability, Li et al. designed a hybrid framework named Deepph, which combines transformers (capturing global information and long-range dependencies) and BiGRUs (capturing temporal information and local dependencies) specifically to distinguish phosphorylation sites of SARS-CoV-2-infected cells [71]. Deepph achieved comparable performance for S/T phosphorylation sites and Y phosphorylation sites, with Acc values exceeding 85% and MCC values around 0.710. On the basis of benchmark phosphorylation data, they investigated the association of SARS-CoV-2 infection phosphorylation with lung cancer genes. Enrichment analysis results uncovered a significantly notable correlation between SARS-CoV-2-infection-induced phosphorylation and signaling pathways associated with cancer and immune inflammation.

Overall, PSPred-ALE demonstrated the potential of self-adaptive embeddings for improving feature representation, while Deepph successfully captured both long-range dependencies and local features through the combination of transformers and BiGRU. However, while these methods improved prediction performance, they have yet to achieve an optimal balance between feature processing and model complexity. In particular, the high computational cost of transformers may limit their practical usability.

(3) Methods based on pretrained protein language models

Li et al. introduced PhosBERT, based on the ProtBERT model, to identify phosphorylation sites in SARS-CoV-2-infected human cells on the basis of a self-supervised learning strategy [62]. Independent dataset results showed that PhosBERT achieved an AUC of 0.896 for phosphorylated S/T sites while achieving consistent excellent performance for Y phosphorylation sites (AUC = 0.902).

Xu et al. proposed the PTransIPs model by incorporating token and position embedding from BERT, as well as sequence and structural embeddings from ProtTrans and EMBER2 [58]. A hybrid network based on CNN and transformer achieved promising predictive performance, producing AUC values of 0.688 and 0.966 for S/T and Y phosphorylation sites, respectively.

The use of pretrained models offers novel approaches for feature representation and prediction in proteomics, showing significant potential in handling complex sequence data. However, the high computational cost and limited domain-specific adaptability remain key challenges for further improvement.

(4) Hybrid architectures combining manual feature extraction with DL

Three prediction tools for phosphorylation sites in SARS-CoV-2-infected host cells, including DE-MHAIPs [72], Res-GCN [73], and GBMPhos [59], were developed by combining manual feature extraction with hybrid DL architectures. Specifically, Wang et al. [72] adopted features such as DDE, CTD, EAAC, AAindex, PseAAC, and BLOSUM62 to represent amino acid sequences. To select the optimal feature set, they fused multi-information through a differential evolution algorithm and selected the best feature subset using the Group LASSO approach (proposed by Yuan et al.) [74]. Their analysis showed that on the S/T (and Y) data, the AUC values increased to 0.920 from 0.898 (and to 0.983 from 0.821) after Group LASSO feature selection, which outperformed GINI, IG, MDS, MI, LASSO, and MRMD feature selection methods. The well-processed data were fed into a combined architecture of a multi-head attention mechanism and LSTM. The DE-MHAIPs achieved AUC values of 0.917 and 0.978 for S/T and Y phosphorylation site identification, respectively.

Huang et al. introduced GBMPhos, which encoded amino acid sequences using one-hot encoding, BLOSUM62, Z-scale, Binary_5bit_type 1, and Binary_5bit_type 2 [59]. The prediction performance of a single category of features and its combinations were compared, and the results demonstrated that the combination of all features achieved the highest Acc, with a value of 85.3%. They defined a hybrid network architecture containing CNN and Bi-GRU. Validation results on the S/T testing dataset demonstrated that GBMPhos exhibited a strong predictive ability, with Acc, MCC, and AUC values of 85.06%, 0.701, and 0.921. It also exhibited exceptional generalizability in classifying Y phosphorylation sites, with Acc, MCC, and AUC values of 90.00%, 0.797, and 0.900.

These methods combine manually extracted domain features (e.g., BLOSUM62 and PseAAC) with DL networks (e.g., LSTM and GCN), achieving strong predictive performance while maintaining a certain level of biological interpretability. However, the complexity and limited scalability of manual feature extraction may hinder their application in high-dimensional and more complex sequence data. Future efforts should focus on integrating more end-to-end automated feature learning.

Overall, current DL models exhibit excellent performance in capturing sequence features and improving prediction accuracy. Pretrained models and hybrid architectures provide a flexible foundation for developing more powerful prediction tools in the future. Moving forward, efforts should focus on developing adaptive deep learning models capable of dynamically adjusting embeddings and network parameters to improve prediction performance and generalization. Furthermore, larger-scale experimental data should be incorporated to validate model reliability and applicability.

## 5. Web Servers and Packages

To provide convenience for researchers in related fields and fully exploit the functionality of algorithms, most prediction models of phosphorylation sites in host cells infected with SARS-CoV-2 have been packaged into computational tools (Table 3). Thereinto, DeepIPs, IPs-GRUAtt, and GBMPhos provide both online (requiring only the input of sequences) and local (facilitating the large-scale use) applications. Additionally, the online website-embedded tool of MeL-STPhos is offered, and the source codes of DE-MHAIPs, PSPred-ALE, PTransIPs, and Res-GCN are available. Users can freely access the corresponding web servers and download packages from the GitHub repository.

## 6. Discussion

In this survey, we have reviewed all the state-of-the-art computational methods for identifying protein phosphorylation sites of SARS-CoV-2-infected human and African green monkey cells. These tools are developed based on the A549, Vero E6, and combined benchmark datasets [26,27]. Two studies have conducted analysis on the influence of window sizes ranging from 5 to 33 on the predictive performance of PSPred-ALE and GBMPhos, respectively. PSPred-ALE performed similarly on the datasets with sequence lengths of 29 and 33 and achieved the highest AUC values of 0.913 and 0.911, respectively. GBMPhos also performed best on the dataset with a window size of 33, with an AUC value of 0.916. Besides, all other tools are developed based on the 33 amino acid datasets. These attempts and assessments may indicate that the optimal window size of phosphorylation sample sequences could be 33 and that the current benchmark datasets are reliable.

The detailed feature encoding and selection strategies, ML models and DL networks, evaluation algorithms and metrics, as well as the specific prediction capability of each tool, have also been summarized. In conclusion, the feature engineering of these ML- and DL-based predictors chiefly focuses on the following strategies: (1) manual informative feature descriptor based on protein sequence, physicochemical properties, evolutionary information, and structure; (2) multiview feature embedding approaches characterizing hierarchical dependencies within biological systems; and (3) a fusion framework that combines various pre-prepared feature information and hybrid deep learning architectures.

Among all predictors, only the MeL-STPhos_2 model is trained for predicting S/T phosphorylation sites in Vero E6 cells infected with SARS-CoV-2, achieving an AUC value of 0.913. Additionally, only the MeL-STPhos_3 model is designed to predict S/T phosphorylation sites in both A549 and Vero E6 cells infected with SARS-CoV-2, but its prediction ability is limited, the accuracy and AUC of which are less than 0.8. All other prediction tools are trained to predict phosphorylation sites in SARS-CoV-2-infected A549 cells. The prediction Acc and AUC values of phosphorylated S/T sites range from 80.63% to 85.49% and from 0.894 to 0.923, which are estimated by 5/10-fold cross-validation. On the Y phosphorylation site dataset, these models achieve higher Acc and AUC values of 83.33~95.24% and 0.900~0.978, respectively. It is worth mentioning that the size of the Y phosphorylation site dataset is much smaller than the S/T site dataset, but better overall prediction performance is obtained on the Y-site dataset. This might imply that although most ML algorithms (especially end-to-end DL methods) perform well with large-scale data, some can also work well in some regions with low data.

Over the past few years, numerous advanced prediction tools have been designed to effectively recognize phosphorylation sites in SARS-CoV-2-infected host cells, which have significantly enhanced our understanding of phosphorylation modification and pave the way for future investigations into the functional characteristics of phosphorylation sites in the SARS-CoV-2-infected host cell. However, several pertinent issues persist for further exploration. For example, the current related computational studies are limited to two available benchmark datasets (namely A549 and Vero E6), and a majority of these methodologies focus solely on analyzing the human A549 datasets. This underscores the need for further search, collection, and expansion of phosphorylation data from diverse host cells infected with SARS-CoV-2 for building more comprehensive and well-defined benchmarks for phosphorylation site prediction. The overall prediction accuracy of S/T phosphorylation sites is just about 85%, which leaves room for improvement. More important and widely used feature extraction and selection algorithms, as well as artificial intelligence models, could be used to greatly increase the computational prediction accuracy of phosphorylation modifications in relation to SARS-CoV-2 infection. To date, few classifiers for identifying phosphorylation sites in host cells infected with SARS-CoV-2 have been implemented based on open-source protein language models (PLMs). Future works should attempt to leverage PLMs or even exploit post-translational modification–specific language models to refine the prediction, generalization, and interpretation capabilities of the computational tools for recognizing phosphorylation sites.

The accurate identification and quantitative analysis of phosphorylation sites are crucial for understanding the infection and regulation mechanisms of SARS-CoV-2. The ML- and DL-based models designed for computationally identifying S/T/Y phosphorylation sites of SARS-CoV-2-infected cells might prompt the discovery of novel antiviral drugs [75]. In summary, we hope that this review can supplement and enrich the knowledge and information related to protein phosphorylation and can provide insights in the exploration of novel computational tools and experimental analysis about SARS-CoV-2-infection-induced phosphorylation modifications.

## Figures and Tables

**Table 1:** The feature representation methods employed to develop SARS-CoV-2-infected phosphorylation site identification tools.

Feature Type	Feature Descriptor (Abbreviation)	Feature Descriptor (Full Name)	Tool
Sequence-based features	AAC	Amino acid composition	EnsembleML, MeL-STPhos
	ANBPB	Adapted normal distribution bi-profile Bayes	Res-GCN
	BINA/BE	Binary profile/binary encoding	MeL-STPhos, Res-GCN
	CKSAAP	Composition of k-spaced amino acid pairs	EnsembleML
	DDE	Dipeptide deviation from expected mean	MeL-STPhos, DE-MHAIPs
	DPC/DC	Dipeptide composition	MeL-STPhos
	EAAC	Enhanced amino acid composition	MeL-STPhos, DE-MHAIPs, Res-GCN
	EGSM	A combination of Shannon entropy, Geary autocorrelation, sequence-order coupling number, and Moran autocorrelation	MeL-STPhos
	One-hot encoding	One-hot encoding	GBMPhos
	QSO	Quasi-sequence-order	MeL-STPhos
Fusion features based on physicochemical properties and sequence composition	188D	A physicochemical property-based 188D feature based on an extended combination of AAC and CTD	EnsembleML
	AAI/AAindex	Amino acid index	MeL-STPhos, DE-MHAIPs, Res-GCN
	ABC	Atomic and bond compositions	MeL-STPhos
	Bit3	Binary 3-bit	MeL-STPhos
	Bit5	Binary 5-bit	MeL-STPhos, GBMPhos
	CKSGP	Composition of k-Spaced amino acid Group Pairs	MeL-STPhos
	CTD	Composition–transition–distribution	EnsembleML, MeL-STPhos, DE-MHAIPs
	EGAAC	Enhanced grouped amino acid composition	MeL-STPhos, LGB-IPs
	EXAC	A linear combination of enhanced AAC (EAAC) and enhanced grouped AAC (EGAAC)	MeL-STPhos
	GXPC	Grouped tripeptide composition	MeL-STPhos
	KSC	K-spaced conjoint triad	MeL-STPhos
	OPF	Overlapping property features	MeL-STPhos
	PseAAC	Pseudo-amino acid composition	DE-MHAIPs, Res-GCN
	XPAAC	A combination of pseudo AAC (PAAC) and amphiphilic PAAC (APAAC)	MeL-STPhos
	ZSC	Z-scale	EnsembleML, MeL-STPhos
Evolutionary-based features	BLOS	BLOSUM62	MeL-STPhos, LGB-IPs, DE-MHAIPs, Res-GCN, GBMPhos
Structural-based features	AESNN	Alignment-based encoding of secondary structure neural network	EnsembleML, MeL-STPhos

**Table 2:** The existing tools for identifying phosphorylation sites in SARS-CoV-2-infected host cells and the specific methods employed in each tool.

Tool	Date	Dataset	Feature Extraction	Feature Selection	**Classifier**	**Model**
DeepIPs	2021	A549	Embedding layer	_	DL	CNN + LSTM
EnsembleML	2022	A549	188D, AESNN3, CKSAAP, zScale	An ensemble learning-based feature selection method	ML	RF
DE-MHAIPs	2023	A549	DDE, CTD, EAAC, AAindex, PseAAC, BLOSUM62	Group LASSO	DL	Multi-head attention mechanism + LSTM
IPs-GRUAtt	2023	A549	Embedding layer	_	DL	BiGRU + Attention mechanism
MeL-STPhos_1	2023	A549	AAC, DPC, AAI, CTDC, CTDT, CTDD, CKSGP, KSC, GXPC, XPAAC, DDE, QSO, EGSM, ABC, AESNN, Bit3, Bit5, OPF, BINA, ZSC, BLOS, EXAC	Tree-based algorithms	ML	Five baseline models (AB, LGBT, CB, GBT, RF) + CB
MeL-STPhos_2	2023	Vero E6	AAC, DPC, AAI, CTDC, CTDT, CTDD, CKSGP, KSC, GXPC, XPAAC, DDE, QSO, EGSM, ABC, AESNN, Bit3, Bit5, OPF, BINA, ZSC, BLOS, EXAC	Tree-based algorithms	ML	Five baseline models (AB, XGBT, GBT, LGBT, CB) + CB
MeL-STPhos_3	2023	Combined	AAC, DPC, AAI, CTDC, CTDT, CTDD, CKSGP, KSC, GXPC, XPAAC, DDE, QSO, EGSM, ABC, AESNN, Bit3, Bit5, OPF, BINA, ZSC, BLOS, EXAC	Tree-based algorithms	ML	Five baseline models (RF, XGBT, AB, LGBT, CB) + CB
PSPred-ALE	2023	A549	Embedding layer	Feature optimization block	DL	Multi-head attention mechanism
Deepph	2024	A549	Embedding layer	_	DL	Transformer + BiGRU
LGB-IPs	2024	A549	EGAAC, BLOS	_	ML	LGBT
PhosBERT	2024	A549	Embedding layer	_	DL	Transformer + A custom DL architecture
PTransIPs	2024	A549	Embedding layer	_	DL	CNN + Transformer
Res-GCN	2024	A549	BE, ANBPB, EAAC, DC, AAindex, PseAAC, BLOSUM62, Word2Vec	Elastic net	DL	GCN + ResNet
GBMPhos	2024	A549	one-hot encoding, BLOSUM62, ZScale, Binary_5bit_type 1, Binary_5bit_type 2	_	DL	CNN + BiGRU

**Table 3:** The independent testing performance of each SARS-CoV-2-infected phosphorylation site predictor on validation datasets and the accessible websites for these tools.

Tool	K-Fold	Acc (%)		MCC		AUC		Github or Web-Server (Accessed on 8 November 2024)
		S/T	Y	S/T	Y	S/T	Y	
DeepIPs	5	80.63	83.33	0.632	0.718	0.894	0.925	https://github.com/linDing-group/DeepIPs http://lin-group.cn/server/DeepIPs/
EnsembleML	_	80.81	95.24	_	_	_	_	_
DE-MHAIPs	5	83.71	91.40	0.675	0.838	0.917	0.978	https://github.com/YuBinLab-QUST/DE-MHAIPs/
IPs-GRUAtt	5	84.62	92.86	0.632	0.858	0.919	0.921	https://github.com/GreatChenLab/IPs-GRUAtt https://cbcb.cdutcm.edu.cn/phosphory/
MeL-STPhos_1	10	83.00	_	0.661	_	0.912	_	https://balalab-skku.org/MeL-STPhos/
MeL-STPhos_2	10	83.60	_	0.673	_	0.913	_	https://balalab-skku.org/MeL-STPhos/
MeL-STPhos_3	10	70.10	_	0.405	_	0.763	_	https://balalab-skku.org/MeL-STPhos/
PSPred-ALE	5	83.14	_	0.663	_	0.907	_	https://github.com/jiaoshihu/PSPred-ALE
Deepph	_	85.49	85.18	0.71	0.705	_	_	_
LGB-IPs	10	82.60		0.653		0.905		_
PhosBERT	5	81.90	87.10	0.6879	0.8581	0.896	0.902	_
PTransIPs	5	84.38	92.68	0.6879	0.8581	0.923	0.966	https://github.com/StatXzy7/PTransIPs
Res-GCN	5	83.50	88.10	0.673	0.763	0.913	0.977	https://github.com/YuBinLab-QUST/Res-GCN/
GBMPhos	_	85.06	90.00	0.701	0.797	0.921	0.9	https://github.com/Xiaorunjuan0405/GBMPhos http://www.biolscience.cn/GBPhospred/
